# Clinical Characteristics and Treatment Outcomes in Endogenous Cushing's Syndrome: A 15-Year Experience from Thailand

**DOI:** 10.1155/2020/2946868

**Published:** 2020-03-12

**Authors:** Wasita Warachit Parksook, Nitchakarn Laichuthai, Sarat Sunthornyothin

**Affiliations:** ^1^Division of General Internal Medicine, Department of Medicine, Faculty of Medicine, Chulalongkorn University, and King Chulalongkorn Memorial Hospital, Thai Red Cross Society, Patumwan, Bangkok 10330, Thailand; ^2^Division of Endocrinology and Metabolism, Department of Medicine, and Hormonal and Metabolic Research Unit, Excellence Center in Diabetes, Hormone and Metabolism, Faculty of Medicine, Chulalongkorn University, and King Chulalongkorn Memorial Hospital, Thai Red Cross Society, Patumwan, Bangkok 10330, Thailand

## Abstract

The most common subtype of endogenous Cushing's syndrome (CS) is Cushing's disease (CD), with higher proportions of adrenal CS reported from Asia, compared to other continents. However, little was known about CS in this territory. This study was to investigate the distribution, clinical characteristics, and treatment outcomes of CS in a single tertiary hospital in Thailand. We performed a retrospective evaluation of 82 patients with endogenous CS during 2001–2015. The most common subtype was CD, followed by adrenal CS and ectopic ACTH syndrome (EAS), respectively. Weight gain was the most common presentation. Normal body mass index (BMI), Asian cutoff, was observed in 33% of patients. Specific features of CS (plethora, muscle weakness, bruising, and/or wide purplish striae) were documented in less than half of patients. The median age, adrenocorticotropic hormone (ACTH), and urinary free cortisol (UFC) concentrations were significantly different among 3 subtypes of CS and were highest among patients with EAS. An initial remission rate after transsphenoidal surgeries in CD was 62%, with higher rates in pituitary microadenomas compared to macroadenomas. All patients with unilateral adrenal disease achieved CS remission after adrenal surgeries. Patients with EAS achieved CS remission mostly from bilateral adrenalectomy. The highest mortality rate was observed in the EAS group. These findings were consistent with previous studies in Asia, with more proportions ACTH-independent CS.

## 1. Introduction

Cushing's syndrome (CS) is a state of excessive endogenous cortisol secretion. It is rare, with an estimated prevalence of 40 cases per million and an incidence of 0.7–2.4 cases per million per year [1–3]. It is more common in women and can occur at any age, even though it tends to occur during the fourth to sixth decades of life [1–3]. Worldwide, the most common cause of CS is Cushing's disease (CD), followed by adrenal CS and ectopic ACTH syndrome (EAS) [1–4]. CS is associated with deleterious effects to health [5]. A 5-year mortality rate in active CS was around 50% due to infection and cardiovascular complications in 1952 [6]. In 1979, mortality rates markedly decreased due to combination treatment [7]. Restoring eucortisolism leads to clinical and biochemical improvement regarding metabolic disturbances, bone health, immune dysfunction, hypercoagulable state, and quality of life.

Studies in Asia showed higher distribution of adrenal CS, ranging from 20–75% of CS etiology [8–12]. Outcomes of CS treatment varied among subtypes and studies. There was only one small case series from the region of Southeast Asia [10]. To date, little is known regarding CS in this region. Therefore, our primary objective is to investigate the distribution of CS in a single tertiary hospital in Thailand. Secondary objectives are to investigate clinical presentations, management, and treatment outcomes of CS in our center.

## 2. Materials and Methods

We performed a retrospective study in a tertiary referral hospital, King Chulalongkorn Memorial Hospital (KCMH), Bangkok, Thailand. All patients aged 18 years and over with the diagnosis of CS between the year 2001 and 2015 were included, using the ICD-10 codes for CS (E24). Description synonyms were adrenal CS, CD, CS myopathy, hypercortisolism, pituitary-dependent hypercortisolism, and pituitary-dependent CD. Diagnostic criteria that suggest CS were urinary free cortisol (UFC) concentration greater than the normal range for the assay, serum cortisol greater than 50 nmol/L after an overnight/low-dose dexamethasone suppression test (DST), and/or elevated late-night salivary cortisol (LNSC) [13]. Patients with a history of exogenous steroid use were excluded. This study protocol was approved by the local ethics committee.

### 2.1. Definitions

Patients with blood pressure from 140/90 mmHg, self-reported history of hypertension or taking antihypertensive agents were classified as hypertensive. Diabetes mellitus (DM) was diagnosed according to the guideline [14]. Patients with fasting plasma glucose (FPG) ≥7.0 mmol/L or HbA1C ≥6.5% or 2-hour glucose ≥11.1 mmol/L during oral glucose tolerance test (OGTT) or having treatment with antidiabetic medications were classified as diabetes. Impair fasting glucose (IFG) was defined if fasting glucose ranged from 5.6–6.9 mmol/L. Impaired glucose tolerance (IGT) was defined if a 2-hour glucose during OGTT was 7.8–11.0 mmol/L. Dyslipidemia was defined if triglyceride (TG) ≥1.7 mmol/L or low density lipoprotein (LDL) ≥3.4 mmol/L or high density lipoprotein (HDL) <1.0 mmol/L in men or <1.3 mmol/L in women or total cholesterol (TC) ≥5.2 mmol/L or having treatment with lipid-lowering agents. Venous thromboembolism (VTE) was defined as having an evidence of clot or thrombosis at any sites proven by relevant imaging. Normal body weight was classified as body mass index (BMI) of less than 23 kg/m^2^ according to the World Health Organization (WHO) classification for Asians. Remission from CS was considered in only patients with both clinical and biochemical remission. Clinical remission of CS was defined by the disappearance of Cushing's stigmata (wide purplish striae, proximal muscle weakness, plethora, or bruising). Biochemical remission was defined as the need for glucocorticoid replacement or achieving normalization of dexamethasone suppression serum cortisol (<50 nmol/L) and/or UFC excretion (<414 nmol/day). Recurrence was defined if patient developed clinical signs and symptoms of overt CS after any previous remission, as well as UFC greater than the normal range for the assay, serum cortisol greater than 50 nmol/L after an overnight/low-dose DST, and/or elevated LNSC. Persistent CS was failure to demonstrate remission after treatment.

### 2.2. Biochemical Measurements

ACTH and serum cortisol samples were collected between 7 and 9 AM and were measured with chemiluminescent immunometric assay (Immulite/Siemens). UFC was measured with radioimmunoassay (Immulite/Siemens). All other biochemical variables were assayed in the central laboratory of KCMH, using standardized analytical methods.

### 2.3. Preoperative Imaging Analysis and/or Procedures

Magnetic resonance imaging (MRI) of pituitary gland was done in all patients with CD. A microadenoma was defined as an adenoma sized less than 1 cm. A macroadenoma was defined as an adenoma sized from or greater than 1 cm. Adrenal imaging using either computed tomography (CT) or MRI was done in all patients with adrenal CS.

Patients with EAS underwent CT scans of the neck, chest, abdomen, and pelvis or octreotide scans. Inferior petrosal sinus sampling (IPSS) and/or high-dose dexamethasone suppression test (HDDST) were done in patients with ACTH-dependent CS with inconclusive pituitary imaging.

### 2.4. Treatment

All procedures were performed by physicians in KCMH. Patients with CD underwent transsphenoidal surgery (TSS) with selective adenomectomy by neurosurgeons. Biochemical remission was defined as postoperative cortisol <138 nmol/L within 7 days after the surgery. Delayed remission was defined as hypocortisolemia (morning cortisol <138 nmol/L) or achieving normalization of dexamethasone suppression serum cortisol (<50 nmol/L) and/or UFC excretion (<414 nmol/day) within 30 days after the surgery. Patients with either persistent or recurrent Cushing's after initial TSS received second-line therapeutic options (surgery, medications, and/or radiotherapy). Patients with adrenal CS underwent unilateral adrenalectomy by urologists, followed by glucocorticoid replacement in unilateral cortisol-secreting adrenal adenomas. Subsequent adrenalectomy was done in patients with bilateral diseases and had persistent hypercortisolism. Patients with EAS underwent surgical removal if tumor localizations were successful. Patients with occult or metastatic EAS were treated with either medications or bilateral adrenalectomy as initial managements.

### 2.5. Statistical Analysis

Since the data were nonnormally distributed, continuous variables were presented as medians with interquartile range (IQR). Group comparisons were examined with Kruskal–Wallis test for nonparametric testing or Chi-squared tests. A *p* value of less than 0.05 was considered to be statistically significant. Statistical analysis was performed with SPSS statistics version 22.0.

## 3. Results

### 3.1. Subtypes of CS

There were 82 patients diagnosed with CS. There were 58 patients with unsuppressed DST, while the rest had UFC excretion greater than the normal range. None of our patients were diagnosed using LNSC because the test was unavailable at our institution at that time. None of our patients were taking medications that could affect the hypothalamic-pituitary-adrenal (HPA) axis at the time of testing. These medications included strong CYP3A4 inducers or inhibitors, oral contraceptive pills, and medical therapies used in clinical practice for the treatment of CS. The most common subtype was CD (45%), followed by adrenal CS (32%) and EAS (13%). All patients with CD had benign ACTH-producing pituitary adenomas. For adrenal CS, the most common etiology was unilateral cortisol-producing adrenal adenoma (62%). Malignant adrenal tumors accounted for 15% of adrenal CS. Primary tumors in patients with EAS comprised both benign and malignant process. Almost half of primary tumors (45%) originated from thorax (Table 1).

### 3.2. Clinical Features

**Figure 1:**
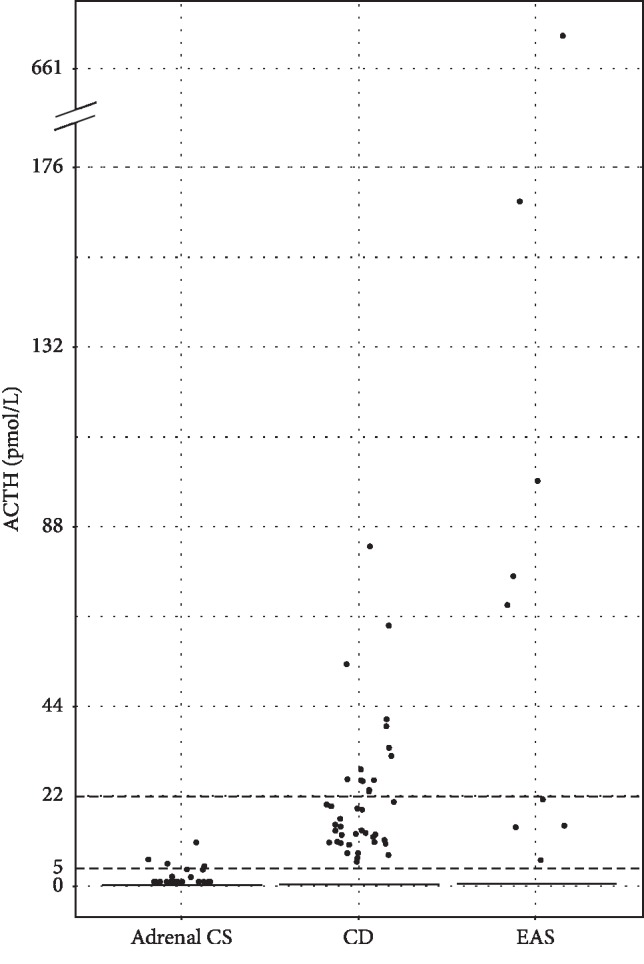
Scatter plots of the median ACTH concentrations. ACTH, adrenocorticotropic hormone; CS, Cushing's syndrome; CD, Cushing's disease; and EAS, ectopic ACTH syndrome.

Weight gain was the most common presentation (Table 2). Moon face and/or supraclavicular fullness were present in most patients, while more specific features of CS (facial plethora, wide purplish striae, easy bruising, or proximal muscle weakness) were documented in less than half of patients. Of the 12 CD patients with available data on sex hormones prior to surgery, 4 patients had hypogonadotrophic hypogonadism. There were no CD patients with preoperative central hypothyroidism; 32 patients had normal thyroid function tests, and none had preoperative thyroid hormone supplement. The median age, ACTH, and UFC concentrations were significantly different among 3 subtypes of CS. These parameters were highest among patients with EAS. Duration between onset of symptoms to the diagnosis of CS and follow-up time was shortest among EAS, but was not significantly different compared with CD and adrenal CS (Table 3). ACTH concentrations were highest in the EAS group (Figure 1). The median ACTH concentrations were 68.9 (IQR 14.8–133.7), 15.0 (IQR 10.8–26.0) pmol/L in patients with EAS and CD, respectively. Most patients (62%) with adrenal CS had ACTH level below 2.2 pmol/L. Four patients with ACTH level greater than 4.4 pmol/L had apparent adrenal diseases and developed postoperative adrenal insufficiency.

### 3.3. Treatment

In CD, an overall remission rate after initial transsphenoidal surgeries was 62% (Table 4). ACTH-positive adenomas were confirmed in 31 patients, while Crooke's hyaline changes were documented in 3 patients. Normal pituitary tissue was documented in 9 patients, and 7 of them had an initial remission after TSS. Patients with pituitary microadenomas had better remission rates which were 68%, compared to 45% in patients with macroadenomas. The median time to recurrence was 23 months (range 12–39 months), with an overall recurrence rate after an initial remission of 13%. The median time to recovery of the HPA axis after TSS was 14 months (IQR 6–24); 10 months (range 6–17 months) in patients with recurrence, and 18 months (IQR 7–27) in the patients without recurrence). In patients with recurrence or persistent CD, 12 patients underwent repeat TSS. Medications were used prior to the subsequent TSS in 8 patients, which were ketoconazole, pasireotide, and mitotane. Nonfatal hepatitis occurred in 1 patient using ketoconazole. Bilateral adrenalectomy was done in 2 patients. There was no report of Nelson's syndrome in both patients during an 80-month follow-up time. Postoperative complications of TSS were found in 30 patients (67%); the most common complications were hypopituitarism (46%), diabetes insipidus (42%), and cerebrospinal fluid leakage (27%). These complications occurred after the first TSS in 25 patients. There was neither report of adrenal crisis nor venous thromboembolism.

All patients with unilateral cortisol-producing, aldosterone- and cortisol-cosecreting and ACC had postoperative adrenal insufficiency, while 3 out of 4 patients with either PPNAD or PBMAH underwent second contralateral adrenal surgery within 1 year after the first surgery due to persistent CS. Among patients with postoperative adrenal insufficiency, 8 patients had been followed up at our institute, and the median time to recovery of the HPA axis was 18 months (IQR 12–25). In patients with EAS, CS remission was achieved in 5 patients (45%), mostly from bilateral adrenalectomy (Table 5). There was only 1 patient who was cured after bronchial carcinoid resection. During a median follow-up of 5 months, 4 patients (36%) died, and 5 patients (45%) lost follow-up while having persistent CS.

## 4. Discussion

We reported a large and comprehensive study on CS in Southeast Asia. The most common subtypes of CS were CD, followed by adrenal CS and EAS. It was more common in female (80%). Usual presentations of the syndrome were weight gain and metabolic complications regarding hypertension and dyslipidemia. The median age, ACTH, and UFC concentrations were significantly different among 3 different subtypes of CS, all of which were highest in patients with EAS.

Consistent with previous studies, the female was predominant in every subtype of CS, with more proportion of male in patients with EAS as in previous reports [15, 16]. Regarding the distribution of CS, proportions of adrenal CS were comparable with studies from Philippines and China, but not as high as studies from Japan and Taiwan (Table 6) [8–12]. It supports a higher prevalence of ACTH-independent CS compared with data from other continents [17–19]. Adrenal adenomas were the most common etiology of adrenal CS in most studies, including ours. Although Ammini et al. reported a higher number of patients with ACC than adrenal adenomas from a single center in India, the author discussed that referral bias might have explained the findings [8]. With an increase in number of abdominal imaging performed these days, an incidentally-discovered adrenal mass was another presentation of adrenal CS, especially in patients with mild CS. However, all of our patients with adrenal CS had obvious clinical manifestations of cortisol excess.

Compared to other studies, weight gain was also a usual presentation, but 33% of our patients still had normal BMI even by Asian standard according to WHO classification [10, 16, 19, 20]. Therefore, being normal weight was not uncommon for CS in Thailand. Hypertension and dyslipidemia were the most common metabolic derangements. Frequency of these comorbidities was comparable with the previous study [8, 16]. Prevalence of DM in this study was less than previous reports which could have been from different testing procedure [8, 9, 16]. Since we did not perform OGTT in every patient, the number of patients with DM might have been underestimated. Only 20–40% of patients were documented having clinical features with more discriminant index of CS which include facial plethora, easy bruising, proximal muscle weakness, and wide purplish striae. Earlier study reported these signs in 56–88% of patients [20]. We could explain this frequency from incomplete documentation and failure to identify these signs from physical examinations. Asian skin could hardly explain this due to inconsistent results from the Philippines showing the percentage of patients having these clinical signs of 50–80% [10]. Hyperpigmentation, which was reported in around 20% of patients with EAS, was also not reported in our series [15, 21].

Regarding the study in by Reynolds et al., cortisol levels in Asians were lower than Europeans with CS [22]. The median serum cortisol level in our study was 717 nmol/L (IQR 500–1081), and the median urinary free cortisol was 1593 nmol/day (IQR 544–2616). Cortisol levels in our patients were higher than Asians' levels in the mentioned study. However, other studies in Asia, e.g., Ammini et al. and Edward N. Lo et al. demonstrated comparable levels of cortisol to our study.

In adrenal CS, there were 4 patients with ACTH concentrations of greater than 4.4 pmol/L. One patient was diagnosed with carcinoma while having an ACTH level of 10.6 pmol/L before undergoing adrenal surgery. Since adrenal insufficiency occurred after unilateral adrenalectomy in all patients with detectable ACTH. ACTH assay interference is the most likely explanation for this finding.

The median remission rate after an initial TSS in CD was 79% (range 25–100%) from a systematic review which included 6869 patients from different countries, including the studies from Japan and Korea [23, 24]. Higher initial remission and lower recurrence were more common in microadenoma than the macroadenoma subgroup. Compared to studies from Japan and Korea, our remission rate was lower, but had higher proportion of macroadenomas [24, 25]. There was no report of Nelson's syndrome as this specific condition increases with longer follow-up time. Treatment outcome in adrenal CS was better than other subtypes of CS, with most patients had remission after their first surgeries. The median time to recovery of the HPA axis in CD and adrenal CS in our study were comparable with the previous study [26]. Adrenal function in CD patients with recurrence after initial remission recovered earlier than in patients without recurrence. For EAS, 36% of our patients underwent bilateral adrenalectomy. Ilias et al. also reported the same rate of bilateral adrenalectomy of 36% in patients with EAS in the National Institutes of Health (NIH) cohort [15]. Mortality rates were highest among this subtype of CS. It might have been higher since a number of patients were lost to follow-up before cured.

This was the largest study done in the region of Southeast Asia. Our study demonstrated clinical profiles and treatment outcomes in all subtypes of CS. Limitations were due to study design which was retrospective in nature. Moreover, referral bias might have occurred because this study was done in a single tertiary care setting. Characteristics of patients may differ from those in the national study. This requires a further national cohort.

## 5. Conclusions

In conclusion, this study reported clinical characteristics and treatment outcomes of CS in a tertiary referral center in Thailand. The distribution of CS subtypes was the same as previous studies in Asia, with more proportions of patients with ACTH-independent CS. Common presentations were weight gain, hypertension, and dyslipidemia. Most patients were obese, but a number of patients still had normal body weight. Remission rate was highest in patients with adrenal CS, compared to CD and EAS. Patients with EAS had the highest mortality rate and proportions of having bilateral adrenalectomy to alleviate severe hypercortisolism. However, a multicenter study is needed to better understand the natural history and treatment outcomes of CS in Thailand.

## Figures and Tables

**Table 1:** Subtypes of CS.

Etiology	*n* (%)
CD	45 (55)
Pituitary microadenoma	34
Pituitary macroadenoma	11
Adrenal CS	26 (32)
Cortisol-producing adenoma	16
ACC	4
Aldosterone-and cortisol-cosecreting adenoma	2
PBMAH	2
PPNAD	2
EAS	11 (13)
Pulmonary	6
Small cell lung cancer	2
Carcinoid (bronchial and thymic)	3
Neuroendocrine tumor, unspecified	1
Abdomen	2
Pancreatic neuroendocrine tumor	1
Pheochromocytoma	1
MTC	1
Uncertain	2

CS, Cushing's syndrome; CD, Cushing's disease; ACC, adrenocortical carcinoma; PBMAH, primary bilateral macronodular adrenal hyperplasia; PPNAD, primary pigmented nodular adrenocortical disease; EAS, ectopic ACTH syndrome; and MTC, medullary thyroid carcinoma.

**Table 2:** Clinical features of CS.

Features	Frequency (%)
Overweight and obesity	67
Weight gain	63
BMI <23 kg/m^2^	33
Amenorrhea	30
Moon face	72
Supraclavicular fullness	54
Dorsocervical fat pad	43
Wide purplish striae	37
Proximal muscle weakness	24
Facial plethora	21
Easy bruising	21
Dyslipidemia	73
Hypertension	68
DM	29
IFG	11
Osteoporosis/fracture	9
Low bone mass/osteopenia	6

BMI, body mass index; DM, diabetes mellitus; IFG, impair fasting glucose.

**Table 3:** Clinical characteristics of patients according to different subtypes of CS.

Features	CD (*n* = 45)	Adrenal CS (*n* = 26)	EAS (*n* = 11)	*p* value
Age (years)	33 (28–43)	40 (29–53)	48 (45–64)	0.003
Female (*n*, %)	37 (82)	22 (85)	7 (64)	0.308
Onset of symptoms (months)	12 (7–24)	12 (5–24)	7 (3–12)	0.427
Hypertension (*n*, %)	29 (64)	20 (77)	7 (64)	0.519
DM (*n*, %)	14 (31)	7 (27)	3 (27)	0.921
VTE (*n*, %)	1 (2)	1 (4)	2 (18)	0.085
Hypokalemia (*n*, %)	9 (20)	9 (35)	5 (45)	0.149
Morning cortisol (nmol/L)	690 (497–828)	690 (469–745)	1269 (1131–1738)	0.052
UFC (nmol/day)	1593 (759–2616)	378 (113–1673)	3003 (2089–13794)	0.013
Follow-up time (months)	42 (18–75)	32 (11–57)	5 (2–65)	0.174

Data were expressed in median (interquartile range). CS, Cushing's syndrome; CD, Cushing's disease; EAS, ectopic ACTH syndrome; DM, diabetes mellitus; VTE, venous thromboembolism; UFC, urinary free cortisol.

**Table 4:** Treatment outcome in patients with CD.

	Outcome after initial TSS with selective adenomectomy			Remission after multiple surgeries and/or radiation (*n*)
	Remission (*n*)	Recurrent (*n*)	Persistent (*n*)	
Microadenomas (*n* = 34)	23	4	11	6
Macroadenomas (*n* = 11)	5	2	5	1

CD, Cushing's disease; TSS, transsphenoidal surgery.

**Table 5:** Clinical characteristics and treatment outcome in patients with ectopic ACTH syndrome.

No.	Age	Sex	Primary site	Metastasis	MRI	IPSS	CT	Medication	Outcome	Follow-up (months)
1	77	F	NA	—	—	+	—	Ketoconazole	Bilateral ADX	65
2	46	F	NA	—	+	+	—	Ketoconazole	Failure	2
3	47	F	Bronchial carcinoid	—	—	+	+	Ketoconazole	Cured	124
4	39	F	Thymic carcinoid	—		+	—	—	Bilateral ADX	125
5	35	F	Pancreatic NET	Liver	+	+	+	—	NA	5
6	51	M	Small cell lung cancer	Liver, adrenal		NA	—	Ketoconazole	Bilateral ADX	33
7	48	M	Medullary thyroid carcinoma	Liver, lung		—	—	Ketoconazole	Bilateral ADX	1
8	44	M	Small cell lung cancer	Liver		—	+	—	Death	3
9	75	M	NET, unspecified	Liver		—	—	—	Death	NA
10	52	F	Bronchial carcinoid	—	+	—	+	Ketoconazole	Death	NA
11	77	F	Pheochromocytoma	—		—	—	Etomidate	Death	1

F, female; M, male; NA, nonspecified; MRI, magnetic resonance imaging; IPSS, inferior petrosal sinus sampling; CT, computed tomography; ADX, adrenalectomy; AI, adrenal insufficiency; NET, neuroendocrine tumor.

**Table 6:** Subtypes of CS and gender distribution: comparison with published studies.

Studies	Number of patients	Female (*n*, %)	CD (*n*, %)	Adrenal CS (*n*, %)	EAS (*n*, %)	Uncertain (*n*, %)
Orth [17] (USA, 1995)	630	NA	428 (68)	120 (19)	76 (12)	—
Imai et al. [11] (Japan, 1996)	122	99 (79)	30 (25)	88 (72)	4 (3)	—
Invitti et al. [18] (Italy, 1999)	426	345 (76)	288 (67)	113 (26)	25 (6)	—
Valassi et al. [19] (European registry, 2011)	481	390 (81)	317 (66)	130 (27)	24 (5)	10 (2)
Lo et al. [10] (Philipines, 2013)	19	17 (89)	8 (42)	7 (37)	4 (21)	—
Tung et al. [12] (Taiwan, 2013)	84	62 (78)	18 (21)	63 (75)	3 (4)	—
Ammini et al. [8] (India, 2018)	364	250 (69)	215 (59)	71 (20)	22 (6)	56 (15)
Zhou et al. [9] (China, 2019)	1652	1289 (78)	1040 (63)	494 (30)	98 (6)	20 (1)
This study	82	65 (79)	45 (55)	26 (32)	11 (13)	—

CS, Cushing's syndrome; CD, Cushing's disease; EAS, ectopic ACTH syndrome.
